# Silk Fibroin Aggregates at the Air–Water Interface: Amyloid-like Fibrils vs. Self-Assembled Networks

**DOI:** 10.3390/ijms27083546

**Published:** 2026-04-16

**Authors:** Olga Y. Milyaeva, Anastasiya R. Rafikova, Alina S. Koneva, Reinhard Miller, Giuseppe Loglio, Boris A. Noskov

**Affiliations:** 1 Department of Colloid Chemistry, St. Petersburg State University, Universitetsky pr. 26, 198504 St. Petersburg, Russia; nastya.rafikova.2000@mail.ru (A.R.R.); a.koneva@spbu.ru (A.S.K.); b.noskov@spbu.ru (B.A.N.); 2 Institute of Condensed Matter Physics, Technische Universität Darmstadt, D-64289 Darmstadt, Germany; reinhard.miller@pkm.tu-darmstadt.de; 3 Institute of Condensed Matter Chemistry and Technologies for Energy, 16149 Genoa, Italy; giuseppe.loglio@ge.icmate.cnr.it

**Keywords:** silk fibroin, air–water interface, fibrils, spread layers, adsorption layers, dilational surface visco-elasticity, surface ellipsometry, AFM

## Abstract

The dynamic properties of spread and adsorbed layers of amyloid-like silk fibroin fibrils (ALF) differ significantly from the properties of native protein layers (RSF). In the former case, the dynamic dilational surface elasticity and the steady-state adsorbed amount are considerably lower than in the latter case. This high dynamic elasticity of RSF layers is close to that of the layers of solid nanoparticles and is provided by the spontaneous formation of various interconnected supramolecular structures at the interface. The ALF produced at elevated temperatures is also intertwined at the interface but does not form a continuous network. In this case, the layer properties are close to those of the layers of amyloid fibrils of globular proteins. If the ALF dispersion is purified from admixtures of unreacted protein molecules, the dynamic surface elasticity reaches about 140 mN/m, similar to the results for dispersions of amyloid fibrils of globular proteins. The admixtures of unreacted protein molecules of high surface activity significantly influence the dynamic surface properties participating in the self-assembly, thereby leading to a slight increase in the surface elasticity. At the same time, the ALF acts as an effective inhibitor of the formation of supramolecular structures in the surface layer for mixed systems. Under the influence of amyloid fibrils, neither the impurities nor the addition of native RSF lead to mechanical surface properties close to those of native fibroin systems.

## 1. Introduction

Bio-inspired technologies borrow the key principles of self-assembly and hierarchical organization from nature with the aim of creating new materials [1,2,3]. This enables the production of materials with complex structures, possessing an exceptional combination of strength and lightness, and the ability to respond “intelligently” to environmental changes. A typical example is the application of silk fibroin to create multifunctional materials for biomedicine, tissue engineering, cosmetics, bioelectronics, and 3D bioprinting [2,4,5,6,7].

The variety of silk fibroin-based materials is largely due to the protein’s propensity to aggregate as a result of a specific sequence of amino acid residues. Hydrophobic domains in fibroin (for example, the repeating sequences GAGAGS or GAGAGY/GAGAGVGY) are prone to the formation of β-sheets due to hydrogen bonds [8,9]. This feature leads to the formation of two main crystalline structures of the protein, Silk I and Silk II, and a rare Silk III [10,11]. Silk I is a soluble form where β-turns and “disordered” structure elements play the main role. When Silk I is exposed to mechanical stress (stretching or shear), elevated temperatures, or treatment by alcohols (methanol, ethanol), or acids, it transforms into an insoluble form of Silk II, which can be described as a β-sheet-rich state [12,13,14]. This transition is a key event in both the spinning of silk fibers in nature and the weaving of artificial silk-based materials [15,16]. Their elasticity and mechanical strength are mainly caused by a number of arrangements of β-sheet crystals and amorphous regions. In this case, β-strands and β-sheets are aligned along the main fibril axis. Such structures provide Young’s moduli of 2.5 GPa for regenerated silk fibrils. If the conditions promote a more ordered alignment of β-sheets, the formation of fibers with a higher elastic modulus becomes possible [17]. The number of β-crystalline regions also increases when collagen, chitosan, some synthetic polymers, or surfactants are added [18,19].

Amyloid fibrils are another example of protein supramolecular systems, where β-sheets stabilized by hydrogen bonds are the main building blocks [20,21]. Unlike silk fibrils, β-strands in these aggregates are oriented perpendicular to the protofilament axis, with the β-sheets arranged parallel to it [21,22]. The difference in orientation allows obtaining hybrid materials with tunable properties by a combination of two types of β-sheet-rich fibrils, amyloid-type and fibroin-type ones [22]. Note that different aggregates of silk fibroin can be called fibrils. This protein can form thread-like aggregates with different organizations and scales, from macroscopic to nanoscale. This study focuses on amyloid-like fibrils formed by silk fibroin under thermal treatment. On the basis of the aggregation kinetics, it was suggested that the secondary nucleation process dominates the formation of silk fibroin fibrils [23,24]. This feature brings silk fibroin fibrils closer to amyloids and demonstrates the possibility of facilitating the aggregation by the addition of seeds. Other important factors influencing the fibril formation are the same as for the β-sheet transition: pH, temperature, shear rate, influence of interfaces, and concentrations of the protein [13,14,23,25,26,27]. The aggregation of silk fibroin molecules in solutions can be described using an analogy with the formation of surfactant micelles. The hydrophilic parts of the molecules are in contact with water, while the hydrophobic parts are located inside the aggregates [28]. At low protein concentrations, the electrostatic repulsion between charged “micelles” is high enough to provide the system stability. At high concentrations the system becomes unstable leading to the formation of silk fibroin fibrils. Recently a more complex architecture of SF aggregates has been proposed. According to Moreno-Tortolero et al., β-strands stack in a coil-like manner, forming a β-solenoid-like structure instead of “micelles” [14,17].

Adsorption at the liquid surface leads to a local concentration increase and also results in silk fibroin self-assembly [29,30]. Depending on concentration, different types of surface morphology, including two-dimensional networks, branched tree-like structures, and thick layers consisting of numerous filaments, can be observed. Due to the formation of stable and durable interfacial films, silk fibroin can be considered as an alternative to many traditional emulsifiers and surface modification agents in various branches of industry, from pharmacy to the cosmetic industry [6,7,31]. Silk fibroin nanofibrils and nanobrushes can significantly change the surface properties and improve the mechanical properties of layer-by-layer films [18]. Silk fibroin nanofibers and nanobrushes allow the formation of more stable emulsions as compared with non-aggregated silk fibroin molecules and some other peptide nanofibers [31]. Moreover, the surface properties are the key factor determining the interactions of silk fibroin materials with biological media. The possibilities of directed surface functionalization to control a cellular response and molecular recognition are a central challenge in developing the next generation of smart biomaterials and diagnostic systems. The elucidation of how different types of SF supramolecular structures can influence the surface properties can promote the development of this area of materials science.

The changes in mechanical properties induced by 3D or 2D silk fibroin self-assembly can be characterized using the bulk or surface rheology [32,33,34]. The dilatation surface rheology is one of the few techniques that can give new information on the rearrangements of molecules at interfaces. Here, we will step by step distinguish the difference in the behavior of silk fibroin in four different forms. The first one is regenerated silk fibroin (RSF) extracted from Bombix mori cocoons. It is present in the bulk in unbound native form. RSF can undergo self-assembly processes after adsorption at the air–water interface. As a result, the self-assembled structures (SAS), such as networks, thin nano-filaments, and branched ribbons, are spontaneously produced. Another form is the silk fibroin amyloid-like fibrils (ALF) which are obtained by thermal pretreatment of an RSF solution. The heating of the solution at 65 °C for a week results in the formation of ALF—thread-like aggregates with a length of more than 1 µm and a width of about 20 nm. Although fibroin almost does not denature at 65 °C [35,36], it undergoes significant conformational changes at this temperature and produces ALF [23,24].

Since the conversion of RSF to ALF is not 100%, the dispersion after synthesis contains not only the target ALF but also some unreacted protein (UP). The dispersion can be used as it is (unpurified) or ALF can be isolated from UP by centrifugation and redispersed. The obtained dispersion will be denoted below as purified ALF dispersion. Combining different forms of silk fibroin at the interface one can govern the properties of the surface layer. In this study, a combination of dilatation surface rheology, ellipsometry, and atomic force microscopy is used to describe the impact of silk fibroin amyloid-like fibrils on the properties of the air–water interface and trace the differences between them and SF self-assembled structures in this respect.

## 2. Results 

### 2.1. Spread Films of ALF

**Figure 1:**
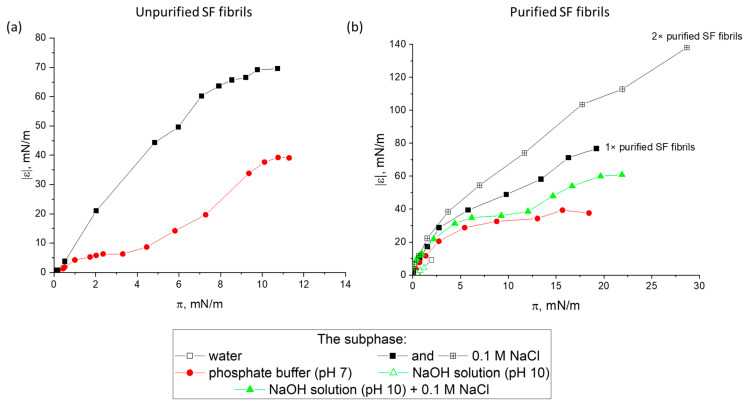
Dependencies of the dynamic elasticity modulus on surface pressure of spread layers of unpurified (**a**) and purified (**b**) ALF on various subphases.

The surface properties of spread protein layers strongly depend on the purification extent and the nature of the subphase (Figure 1). The dynamic surface elasticity increases at the spreading drop-by-drop of the dispersion of unpurified ALF onto the liquid surface (Figure 1a). The surface pressure of about 10 mN/m corresponds to the dynamic surface elasticity of approximately 30 mN/m and 60 mN/m, respectively, if a phosphate buffer or 0.1 M NaCl solution is used as the subphase. Spreading onto a pure water surface did not lead to any noticeable changes in the surface properties.

The crucial role of the subphase is also indicated by the results for spread layers of purified ALF (Figure 1b). In this case, the purified ALF was redispersed at pH 10 to obtain a uniform distribution of aggregates in the dispersion prior to its deposition. The higher pH and the lower ionic strength led to changes in the surface properties. Presumably, the spread ALF can be easily desorbed at pH 10. At the same time, a low ionic strength hinders the spreading of the dispersion along the liquid surface. Such behavior has been previously observed for the spreading of β-lactoglobulin (BLG) and lysozyme fibrils, and for layers of microgel particles of globular proteins [20,37].

**Figure 2:**
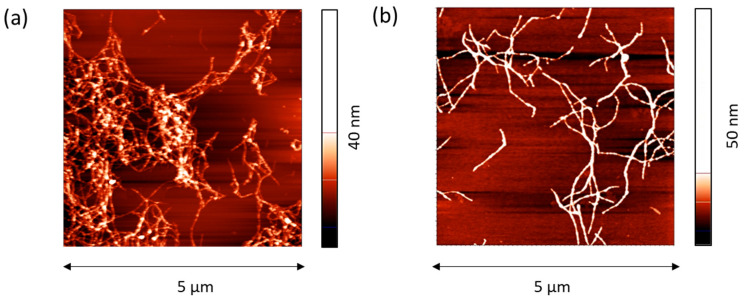
AFM images of spread layers of purified ALF on the surface of 0.1 M NaCl in water (**a**) and NaOH solution at pH 10 (**b**).

The most significant changes occurred if a twice-purified ALF was spread onto the surface of a 0.1 M NaCl solution. In this case, the surface pressure reached about 28 mN/m, corresponding to a dynamic surface elasticity of about 140 mN/m. This value is close to that obtained previously for purified amyloid fibrils of BLG and lysozyme [20]. At the same time, the dynamic surface elasticity of the layers of BLG and lysozyme fibrils was higher than the results for the corresponding native protein layers while for silk fibroin ALF the opposite is true—the surface elasticity is close to or even lower than the values for the layers of RSF. For the latter, the dynamic surface elasticity at the surface pressure of 28 mN/m varies from 130 to 250 mN/m depending on the concentration [29,30]. This effect can be associated with the formation of SAS in the layer of RSF with a large number of β-sheets. Obviously, since the protein in the purified dispersions is predominantly in the form of pre-formed ALF, SAS in the surface layer are not formed. Furthermore, when depositing purified ALF onto the subphase surface, the particles largely remain in an agglomerated state and form on the solution surface separate islands and secondary aggregates (Figure 2). As a result, the high surface pressures can be achieved only by compressing the film by reducing the surface area.

### 2.2. Composite Films of Purified ALF and RSF

**Figure 3:**
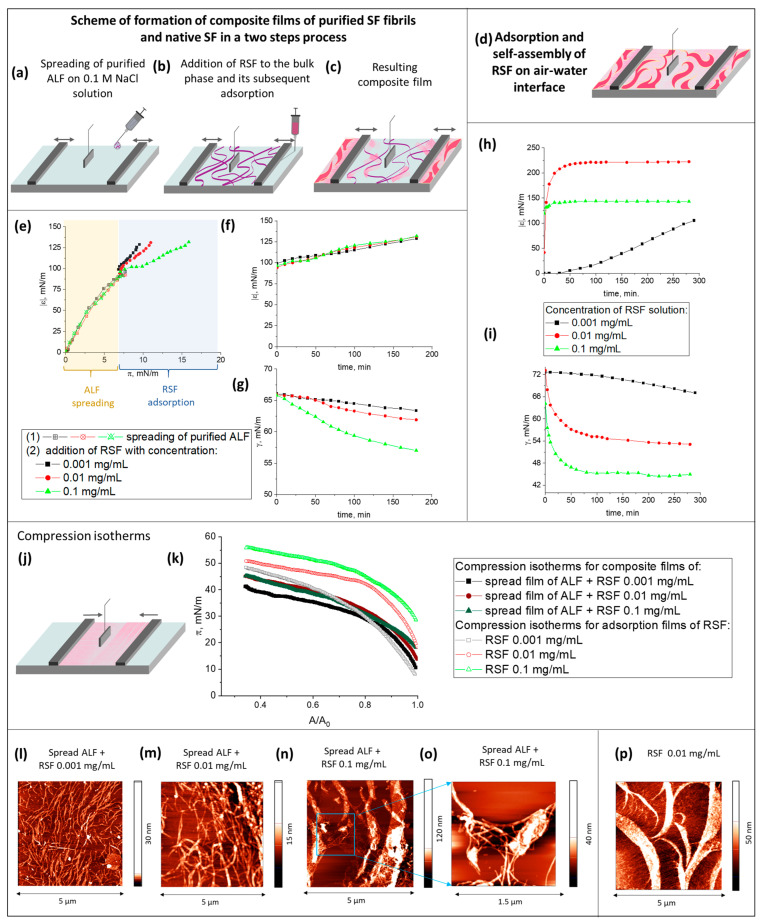
Scheme of formation of composite films (**a**–**c**) and adsorption layers of RSF (**d**). ALF are noted with purple color, SAS—with pink color, arrows indicate the direction of movement of the barriers. The dependencies of the dynamic elasticity modulus on surface pressure for composite films with different concentrations of the protein added to the bulk (**e**). The kinetic dependences of the dynamic surface elasticity (**f**,**h**) and dynamic surface tension (**g**,**i**) for composite films (**f**,**g**) and adsorption layers of native protein (**h**,**i**). Scheme of compression of composite films (**j**). The compression isotherms of the composite films and adsorption layers of native SF (**k**). AFM images of composite layers (**l**–**o**) and the adsorption layer of native protein (**p**).

In order to discover whether the decrease in the dynamic surface elasticity is associated with the presence of ALF or UP in the surface layer, the following experiment was conducted: at the first step, a dispersion of purified ALF was deposited onto the surface of an NaCl solution (Figure 3a–c) until the dynamic surface pressure and surface elasticity reached 8 mN/m and 100 mN/m, respectively (Figure 3e). After that, a solution of RSF was added to the bulk phase, and the subsequent increase in the dynamic surface elasticity and surface pressure due to the adsorption of RSF was monitored (Figure 3e–g). Three concentrations of RSF were chosen for the addition to the subphase in the course of the second step: 0.001, 0.01, and 0.1 mg/L. These concentrations correspond to three different surface structures observed for pure RSF solutions: a two-dimensional network of threadlike aggregates (Figure 3d,p); the coexistence of the two-dimensional network and broader branched ribbons; and a thick film consisting of closely packed thin fibers [30]. To evaluate the influence of the fibril concentration in the surface layer on the formation of SAS, the measurements of surface properties were also carried out with a smaller amount of deposited ALF (Figure S1). ALF was spread until a surface pressure of 3 mN/m and a surface elasticity of 55 mN/m were reached. After that, RSF was added to the bulk phase. Since the results obtained with different amounts of deposited ALF are similar, only the results obtained with the larger deposition volume are discussed hereafter.

One might expect that the formation of a composite fibroin film, consisting of various types of aggregates, would contribute to an increase in the film elasticity and the deposited ALF would act as nucleation sites for the growth of SAS. Furthermore, an increase in the number of β-sheet structures in the surface layer could improve the mechanical properties, as was demonstrated for other fibroin-based materials [12,38,39,40,41]. Meantime, the opposite effect was observed. Despite the addition of RSF leading to an increase in the surface elasticity to approximately 120 mN/m (Figure 3e,f), the obtained values were much lower than those of the RSF layers (Figure 3h). The kinetic dependencies of the dynamic surface elasticity almost coincided despite the hundredfold difference in the concentrations of the RSF (Figure 3f). The dynamic surface tension in the case of the composite surface layer, even for the highest protein concentration of 0.1 mg/mL, reached only 57 mN/m (Figure 3g), while for the RSF solution of the same concentration without ALF, it was 45 mN/m (Figure 3i).

The compression isotherms of the obtained composite films demonstrate a gradual increase in the surface pressure to about 42–45 mN/m (Figure 3j,k). The observed behavior is qualitatively similar to that for adsorption layers of RSF. However, in the latter case it is possible to reach significantly higher surface pressures (Figure 3k). For example, for the concentration of 0.001 mg/mL the compression leads to a more abrupt increase in the surface pressure from 6 to 48 mN/m. In both cases, the density of the layer increases under compression but in the composite film it is more heterogeneous and therefore contains a larger number of local defects.

It is possible to assume that ALF in the surface layer prevents the formation of the SAS observed in the layers of RSF. Although RSF molecules penetrate into the surface layer, their influence on the surface properties turns out to be significantly weaker than in the case of the system without ALF due to major structural differences. AFM results confirm this conclusion (Figure 3l–p). These images show the spread of ALF as long filamentous aggregates, which can be frequently intertwined. The RSF can produce some elements of SAS which can be seen as small inclusions and islands between the fibers (Figure 3n,o). The increase in the RSF concentration leads to the formation of ribbons (Figure 3n). At the same time, these ribbons, unlike the case of adsorption films of pure RSF (Figure 3p), do not form an extended network at the interface. Probably, just this network ensures high values of the elastic modulus of pure RSF solutions. Presumably, an uneven distribution of ALF in the surface layer leads to the formation of heterogeneous surface layers with a relatively low surface elasticity. Therefore, it is possible that the relatively small dynamic elasticity of composite layers discussed in Section 2.1 can be the consequence of an inhomogeneous distribution of ALF in the surface layer.

### 2.3. Adsorption Films of ALF and RSF

#### 2.3.1. Dynamic Surface Elasticity and Surface Tension

**Figure 4:**
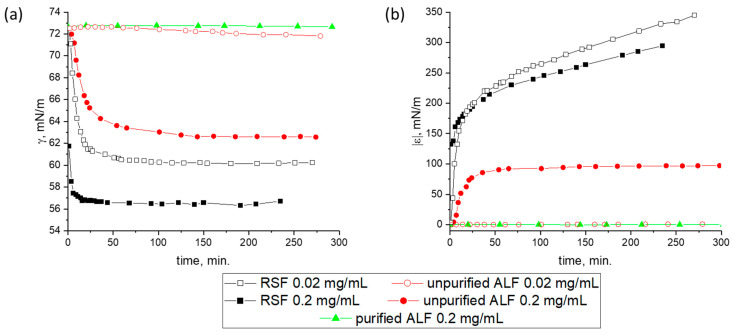
Kinetic dependences of (**a**) dynamic surface tension and (**b**) dynamic surface elasticity of RSF (black squares), unpurified (red circles), and purified (green triangles) ALF dispersions with concentrations of 0.02 mg/mL and 0.2 mg/mL at pH 7.

A more uniform distribution of ALF in the surface layer can be achieved by its adsorption from the bulk phase. The ALF exhibits a significantly lower surface activity as compared with the RSF. The dynamic surface tension and dynamic surface elasticity were measured as a function of surface age, ALF concentration, and the degree of dispersion purification (Figure 4). At a concentration of 0.02 mg/mL, the RSF reduces the dynamic surface tension to 60 mN/m in 30 min after surface formation, while the ALF dispersions, regardless of their purification, almost do not show a noticeable surface activity even after five hours (Figure 4a). Increasing the concentration to 0.2 mg/mL leads to a noticeable surface activity for the dispersion of unpurified ALF and a decrease in surface tension to 63 mN/m. At the same time, for the solutions of purified RSF, a decrease in the surface tension to 60 mN/m is observed only at a concentration of 1 mg/mL. Presumably, the surface tension of unpurified ALF dispersions decreases mainly due to the adsorption of UP. They have a high diffusion coefficient due to their small size as compared to ALF, reach the surface fast, and can participate in the self-assembly processes.

**Figure 5:**
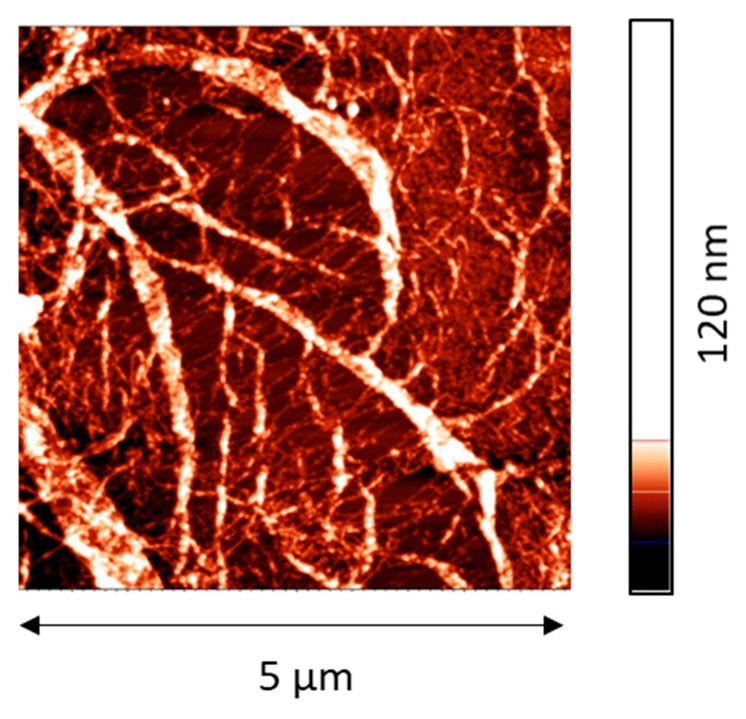
AFM image of the adsorption layer of unpurified ALF with a concentration of 0.02 mg/mL at pH 7.0.

AFM images show that the morphology of the adsorption layer with the SAS in the dispersion of unpurified fibrils at a concentration of 0.2 mg/mL is similar to that previously observed for the RSF layers (Figure 5). The images clearly show broad ribbons and thinner filaments surrounded by an almost two-dimensional network. The thin filaments correspond to incorporated ALF. The significantly lower dynamic surface elasticity as compared to the RSF at similar surface pressures gives indirect evidence of the presence of ALF in the surface layer (Figure 4b).

**Figure 6:**
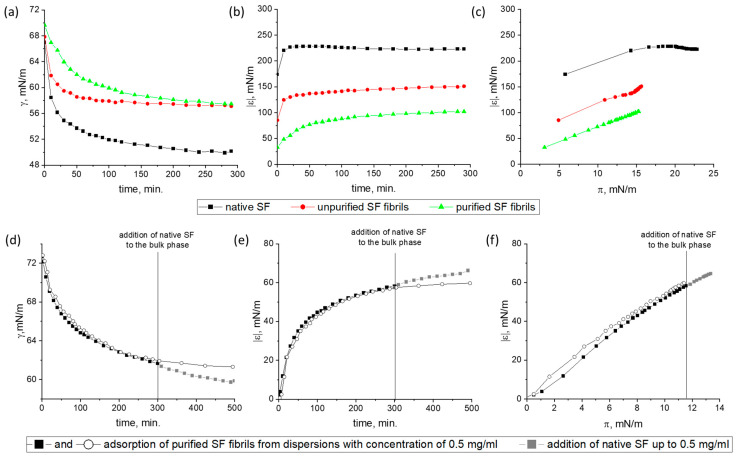
Kinetic dependences of (**a**) dynamic surface tension, (**b**) dynamic surface elasticity, and (**c**) dependence of dynamic surface elasticity on surface pressure of adsorption layers for RSF solutions, unpurified and purified ALF dispersions with a concentration of 1 mg/mL at pH 10.0; (**d**–**f**) data for adsorption layers obtained in dispersions of purified ALF with a concentration of 0.5 mg/mL when the RSF was added to the bulk phase. The concentration of RSF was 0.5 mg/mL and thus the total concentration of silk fibroin in all forms equals 1 mg/mL.

A more detailed comparison of the surface properties of RSF solutions with the results for the dispersions of unpurified and purified ALF was carried out at a concentration of 1 mg/mL. In this case, the purified ALF displays a noticeable surface activity and the dynamic surface elasticity reaches steady-state values within one hour for all systems under study (Figure 6a). At the same time, the dynamic surface tension continues to decrease. The most significant increase in the dynamic surface elasticity, up to 220 mN/m, and the surface pressure, up to approximately 22 mN/m, is observed for the RSF solution (Figure 6a,b), while for unpurified and purified ALF dispersions the dynamic surface elasticity approaches steady state values of about 150 and 100 mN/m, respectively (Figure 6b). The dynamic surface tension of both systems tends towards the same value—about 58 mN/m. At the same surface pressure, the scatter in the surface elasticity for the three systems exceeds 50 mN/m (Figure 6c). Therefore, the decrease in the dynamic surface elasticity is primarily caused by ALF in the surface layer.

The effectiveness of ALF in preventing the formation of SAS in the surface layer can also be confirmed if the adsorption of purified ALF from a solution with a concentration of 0.5 mg/mL is followed after 5 h by the addition of 0.5 mg/mL of RSF to the bulk phase (Figure 6d–f). In this case, the dynamic surface elasticity 2.5 h after the protein addition turns out to be lower than the values not only for the RSF, but even lower than the values for the purified ALF alone (Figure 6e). On one hand this indicates that the adsorption of purified ALF from a solution with a lower concentration is slower and, over five hours, leads to the formation of surface layers with lower elasticity. On the other hand, the addition of RSF does not lead to a noticeable increase in the observed values. Thus, it can be assumed that the fibrils create an additional barrier against the formation of SAS at the interface.

#### 2.3.2. Compression Isotherms

**Figure 7:**
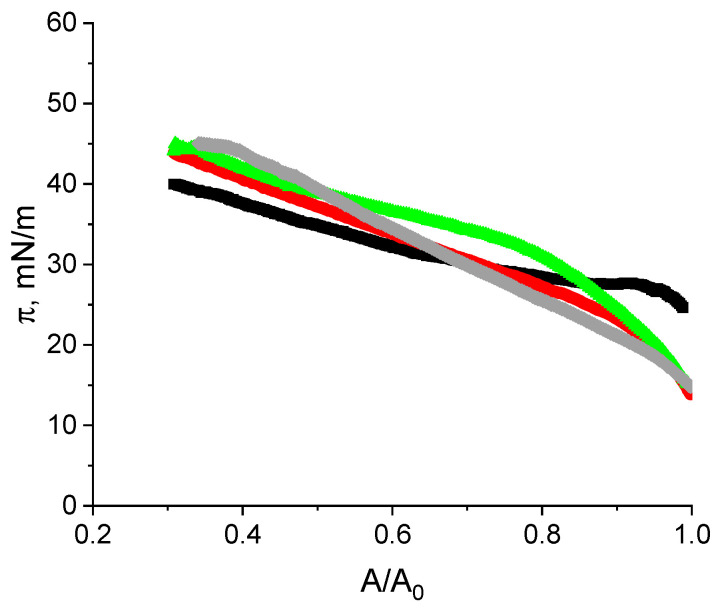
Compression isotherms of SF layers formed from RSF solutions (black line), unpurified ALF dispersion (red line), purified ALF dispersion (green line), and ALF with addition of RSF (gray line). Adsorption layers were obtained at a concentration of 1 mg/mL at pH 10.

The adsorption films of RSF, purified and unpurified ALF and their compositions can be well characterized by compression isotherms (Figure 7). The difference between the isotherms for the layers of RSF and purified ALF is most significant, while the isotherms for composite layers are in between these two cases. For the RSF, the behavior is similar to that described previously [30]. The surface pressure increases gradually, starting from 22 mN/m, and reaches approximately 40 mN/m. Compression of the RSF film causes relatively weak changes in surface pressure due to the significant thickness of the surface layer (cf. Section 2.3.4). For all systems containing ALF the change in surface pressure is more pronounced (from about 15 mN/m to about 46 mN/m). For purified ALF one can distinguish two regions on the compression isotherm corresponding to the different rates of surface pressure change. It can be assumed that under relatively small compression, the fibrils are only partially pushed into the subphase, while under higher compression rates, the aggregates may completely lose contact with the air phase. Such a process requires higher surface stresses compared to the compression of a relatively homogeneous film of native protein.

#### 2.3.3. Lissajous Plots

**Figure 8:**
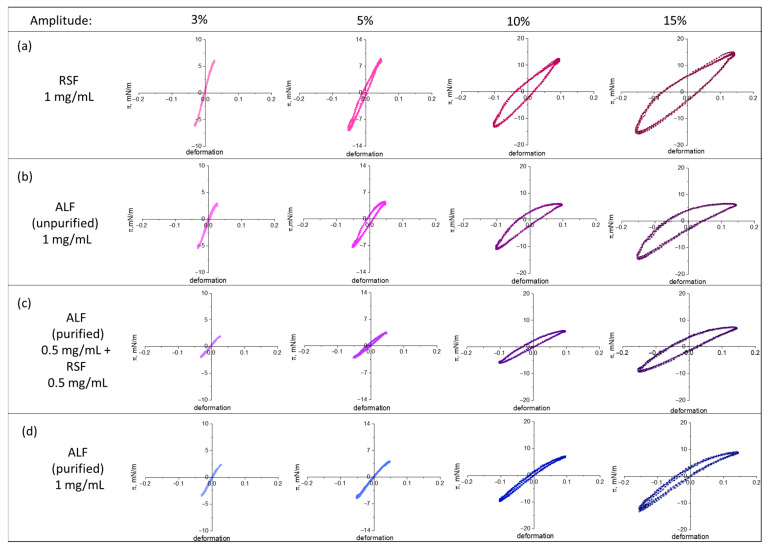
Lissajous plots of surface pressure versus deformation obtained during amplitude sweeps of adsorption layers of RSF (**a**), unpurified ALF (**b**), purified ALF with addition of RSF (**c**), and purified ALF (**d**) at constant protein concentration of 1 mg/mL at pH 10.

Lissajous plots of the surface pressure versus the surface deformation can be used to describe the surface dilatational rheology in terms of the interfacial microstructure (Figure 8). After the equilibrium was established, the adsorption films were subjected to expansions and compressions at various amplitudes. At small oscillation amplitudes, all systems demonstrate an almost linear response. With an increase in the oscillation amplitude, the shape of the plots becomes elliptical, indicating an increase in the viscous contribution to the system response to deformation. For purified ALF, the response remains almost linear even at relatively large deformations (10%), while the strongest deviation from a linear behavior is demonstrated by the adsorption films of RSF and unpurified ALF. For these systems, the Lissajous plots are noticeably asymmetric: the surface pressure upon compression is higher than the surface pressure upon expansion. For RSF, a significant strain hardening upon expansion and a slight strain softening upon compression are observed. For all ALF layers, the situation is reversed: at large amplitudes, the strain softening at expansion and the strain hardening at compression are observed. Narrow Lissajous figures and an increase in their slope with the growing deformation amplitude indicate strain hardening upon compression. It can be assumed that a decrease in the surface area promotes the formation of structured, glass-like or liquid-crystalline-like phases in the surface layer [42]. It can be noted that for similar systems of soy fibrils mixed with polypeptides, and for crude beta-lactoglobulin fibrils, the Lissajous plots practically replicate the behavior of the native protein [43,44]. While in the case of mixed systems with silk ALF, the shape of the curve differs from both RSF and purified ALF. For unpurified ALF the effect of strain softening at expansion and strain hardening at compression was most noticeable. Probably, in this case, the UP acts as an additional structure-forming element, providing a larger proportion of the ordered domains upon compression as compared to other fibrillar systems. Their number decreases upon expansion, and the system exhibits a more viscous behavior. This behavior is in contrast with that of the RSF. At a concentration of 1 mg/mL, RSF forms a thick, highly ordered adsorption layer on the surface, which partially collapses upon compression, but upon expansion restores its structure, consisting of many thin, interconnected fibers.

#### 2.3.4. Ellipsomerty

**Figure 9:**
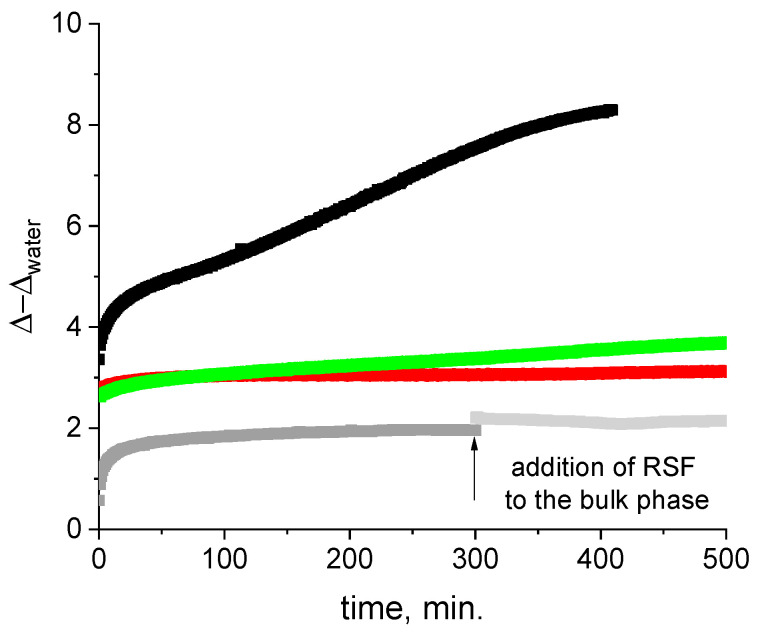
Kinetic dependencies of ellipsometric angle Δ for adsorption layers formed from RSF solutions (black line), unpurified ALF dispersion (red line), purified ALF fibril dispersion (green line), and purified ALF with addition of RSF (gray line). In all cases, the adsorption layers were obtained from solutions with a total protein concentration of 1 mg/mL at pH 10.

The difference between the adsorption layers of RSF and the adsorption layers containing ALF was most pronounced in ellipsometric measurements (Figure 9). The kinetic dependences of the ellipsometric angle Δ show the difference between these systems. For all systems studied, the ellipsometric angle Δ differs significantly from the value for the solvent within the first minutes after surface formation. At the same time, the values of Δ for the RSF layer at this initial step are approximately 1.5 times higher than the values for the systems with ALF. The difference becomes even more noticeable with the increase in surface age and reaches about 5^o^ in 7 h after the start of the measurements. These results allowed estimating the adsorption layer thickness as about 40 nm for RSF and 7–15 nm for systems containing ALF.

It can be assumed that the first adsorption step for both systems is the formation of a monolayer. The next stage of adsorption layer formation for RSF involves the growth of a network of branched thin fibers resulting in the formation of thick layers [30]. In contrast to RSF, the changes in the structure of the ALF adsorption layers after the monolayer formation are smaller and the increase in the delta angle, and therefore of the surface concentration, occurs only due to the adsorption of new portions of ALF and/or the adsorption of free RSF or UP for the composite systems.

#### 2.3.5. Atomic Force Microscopy

**Figure 10:**
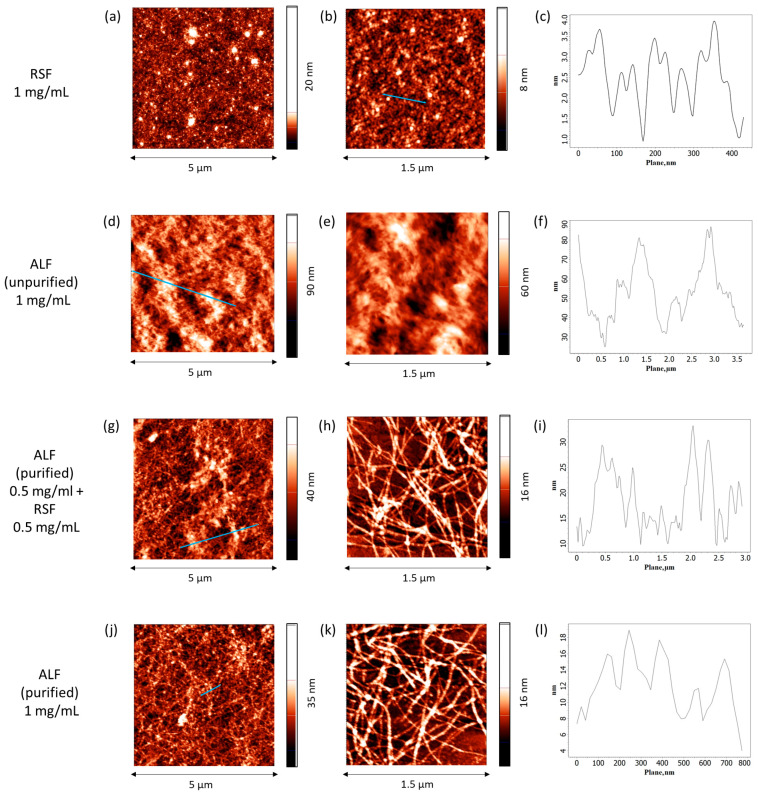
AFM images of spread layers of SF formed from native SF solutions (**a**–**c**), unpurified SF fibril dispersion (**d**–**f**), SF fibrils with the addition of native protein (**g**–**i**), and purified SF fibril dispersion (**j**–**l**). In all cases, the adsorption layers were obtained from solutions with a total protein concentration of 1 mg/mL at pH 10.

AFM results show that the most uniform films with only slight roughness are observed for RSF (Figure 10a,b). Despite the relatively large thickness, the height of the layer changes only slightly along the surface (Figure 10c). RSF undergoes self-assembly at the air–water interface resulting in the formation of many thin, a few nanometers in diameter, densely packed fibers. In the case of unpurified ALF, the individual aggregates are indistinguishable, but their presence can be inferred from the significant height variations (up to 40 nm) (Figure 10d–f). In this image, the fibrils appear to be coated by UP, smoothening the relief as compared to the films of purified ALF. In the latter case, the AFM images clearly show many intertwined fibroin fibrils, more than 1 μm in length and about 20 nm in diameter, packed in 1–2 layers (Figure 10j,k). The films for the system with pre-adsorbed ALF and with the subsequent addition of RSF have a similar morphology (Figure 10g,h), indicating that ALF in the surface layer effectively prevents the formation of SAS.

## 3. Discussion

ALF has a lot of similarity with the amyloid fibrils of globular proteins. Both contain β-sheet structures as the main building blocks and have almost the same morphology. The fibril dispersions are usually used for the stabilization of foams and emulsions without additional purification [31,45,46,47,48,49]. Unpurified dispersions, immediately after the fibril synthesis, contain significant amounts of unreacted protein molecules. In the case of globular proteins, the fibril formation is usually accompanied by the denaturation of the native protein. The produced polypeptides of high surface activity are adsorbed faster than the larger fibrils [20]. In the case of silk fibroin, the moderate heating can cause only a molecular weight decrease [23,24] but does not damage the key peptide sequences, which are responsible for the self-assembly [50]. Therefore, it can be expected that the adsorption of unreacted protein molecules leads to the self-organization.

The produced supramolecular structures provide high stability of the silk fibroin-based disperse systems [6]. A question arises about how different aggregates in the surface layer affect its dynamic properties. Previous studies have shown that the spread and adsorption layers of amyloid fibrils of globular proteins differ greatly in their properties [20]. The influence of unreacted protein impurities is much stronger for the adsorption layers due to a difference in the surface activity between the fibrils and polypeptides. In this case, the main component of the adsorption layers is unreacted protein impurities, and the impact of the fibrils becomes noticeable only at their high concentrations. For the spread layers, this problem is reduced since the concentration of ALF is much higher in the layer. The formation of uniform ALF spread layers is not easy but the uniformity increases under layer compression. In this case, the surface elasticity of the layer of two-times-purified ALF increases to values close to those of amyloid fibrils of globular proteins.

The dynamic elasticity of the unpurified ALF layers is also significantly lower than the values for native RSF layers. It was expected that the fibrils would act as growth centers of the self-assembled structures thereby improving the mechanical properties of the mixed layers compared to RSF layers. Meantime, this did not occur, and the surface elasticity proved to be even lower than that of non-uniform spread layers of purified ALF. This behavior resembles that observed previously for the globular protein layers, where the polypeptides formed after denaturation influenced the surface properties strongly [20]. Nevertheless, measurements of the surface properties of the solutions of UP separated from ALF show that they are close to the properties of the native protein solutions of lower concentrations (Figures S2 and S3) and do not demonstrate any signs of denaturation [51].

A few possible explanations of the low surface elasticity of the layers of unpurified ALF can be proposed. First, the amount of UP could be insufficient to form SAS at the interface. Second, the layers of unpurified ALF are heterogeneous and have a non-uniform distribution of the fibrils. At last, ALF in the surface layer may prevent the formation of SAS leading to a relatively low surface elasticity, much lower than that for the RSF layers.

In order to examine the first possible explanation and to elucidate the effect of ALF on the SAS formation in the surface layer the composite layers of spread purified ALF and adsorbed RSF were studied. It turned out that the mechanical properties of the composite layers are inferior to those of native RSF. Moreover, in the presence of ALF, the supramolecular networks are formed to a significantly lesser extent than in pure RSF layers. It is the formation of SAS in the surface layer that accounts for the unique mechanical properties of RSF-stabilized interfacial layers. In some cases, the RSF-stabilized emulsions can even be considered as Pickering emulsions because of the similarity of SAS to solid nanoparticles [6]. Depending on the concentration, RSF forms various types of supramolecular structures with different responses to deformation [29,30]. If the surface layer contains RSF only, the dynamic surface elasticity is almost five times higher than that of globular protein solutions and comparable with the results for the layers of solid nanoparticles [52]. A possible explanation is a transition from Silk I to helical Silk III or laminated Silk II with a high number of β-sheet crystallites upon adsorption [10,53,54]. Depending on the surface pressure, the fractions of fibroin proportion modifications can vary. The greatest number of β-sheet crystallites corresponds to the surface pressures of 16–17 mN/m [10,53]. Under these pressures, the highest dynamic surface elasticity was observed (Figure 3h,i). The surface pressures for solutions with concentrations above and below 0.01 mg/mL correspond to smaller fractions of β-sheet crystallites and thus to lower surface elasticities. In the meantime, even these values exceed the results for composite films when the surface pressure of 16 mN/m is achieved for the highest concentration of added RSF. AFM images also do not show a continuous SAS network. Therefore, the reduction in surface elasticity for mixed systems is not due to an insufficient RSF concentration, but ALF itself leads to a decrease in the dynamic surface elasticity. In order to exclude the impact of non-uniform distribution of the ALF in the spread layers the surface properties of adsorption films were studied.

The dynamic elasticity of RSF adsorption layers reaches about 230 mN/m, while the corresponding value for purified ALF layers is only about 100 mN/m under the same conditions. The ellipsometry reveals that RSF forms thick layers of approximately 40 nm, whereas ALF layers are much thinner, 7–15 nm. Furthermore, Lissajous plots indicate that RSF layers exhibit strain hardening upon expansion, whereas ALF layers show strain hardening upon compression and strain softening upon expansion (Figure 8), indicating a distinct microstructure. The atomic force microscopy shows that RSF assembles into a uniform, densely packed network of thin fibers, while in the case of ALF, one can observe only intertwined long filamentous aggregates that do not form a continuous network. These findings indicate a role of ALF as a structural modifier that suppresses the interfacial self-organization. Therefore, contrary to expectations, in the mixed adsorption layers (unpurified ALF or ALF with addition of RSF), ALF does not become growth centers for thinner fibers and ribbons, which are typical for RSF layers. It is possible to conclude that a significant number of ALF in the surface layer decreases the available surface area for the formation of supramolecular networks. The change in the dynamic surface properties reflects the change in the adsorption layer structure, leading to a decrease in the dynamic surface elasticity in this case.

## 4. Materials and Methods

### 4.1. Silk Fibroin Isolation

Silk fibroin was extracted from Bombix mori cocoons provided by a Russian domestic farm according to the protocol of Ref. [55]. Briefly, the cocoons were boiled in a 0.02 M Na_2_CO_3_ solution for 40 min. After that, the degummed fibroin was dissolved in a 9.3 M LiBr solution for 4 h at a temperature of 60 °C and dialyzed using a cellulose membrane (Sigma Aldrich, Steinheim, Germany) for 72 h against deionized water. The fibroin solution (1% wt.) was obtained after removing undissolved substances by centrifugation. This regenerated silk fibroin will be further labeled as RSF in order to emphasize the difference from synthesized amyloid-like silk fibroin fibrils (ALF).

### 4.2. Silk Fibroin Fibrils Preparation

ALF was prepared by incubating a 1 mg/mL fibroin solution for 7 days at 65 °C without stirring. The dispersion of fibrils was purified from unreacted protein molecules (UP) by centrifugation (12,500 rpm, 4 °C, 2 h) and subsequent replacement of the supernatant by a NaOH solution (pH 10). The concentration of the purified dispersion was determined by gravimetric analysis.

### 4.3. Thioflavin T Fluorescence Assay

The Thioflavin T (ThT) fluorescence assay was used to confirm the formation of the amyloid fibrils. The kinetics of the ALF formation were determined by measuring the fluorescence intensity of ThT [56]. This dye has a low fluorescence intensity in the free form, but its interaction with the β-sheet structure of fibrillar aggregates leads to an increase in the quantum yield by several orders of magnitude [57]. Ten samples with different incubation times were selected for the study: 0, 0.5, 1, 3.5, 20, 27, 51, 80, 104, and 168 h. Before the measurements, ThT was added to each sample with a concentration of 2 × 10^−5^ M.

**Figure 11:**
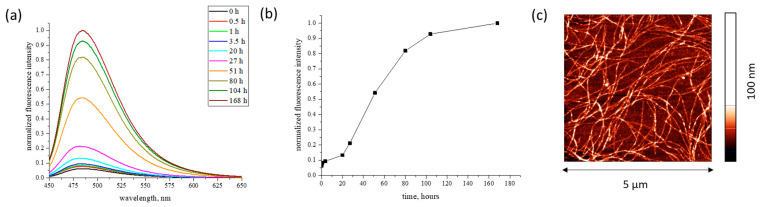
Fluorescence spectra of ThT in the course of SF formation at different incubation times at pH 5.6 and 65 °C (**a**); kinetic dependence of normalized ThT fluorescence intensity (**b**); AFM images of silk fibroin fibrils (**c**).

Fluorescence spectra were obtained in the range of 450–650 nm at an excitation wavelength of 435 nm using a HORIBA spectrofluorometer (Horiba, Kyoto, Japan). The results showed that the ALF formation in water at 65 °C was accompanied by a noticeable induction period of about 20 h (Figure 11a,b), in agreement with the data of [23].

The heating of an RSF solution for 100 h leads to a strong increase in the ThT fluorescence at 480 nm (Figure 11a,b), when the maximum in the excitation spectrum shifts from 412 nm, which is typical for unbound ThT molecules [56,58], to 448 nm (Figure 11a). Such spectral changes are caused by the ThT interactions with an extended β-sheet structure [56,58], confirming the formation of fibrils. The ALFs have an almost uniform morphology and are characterized by a mean length of more than 1 µm and a width of about 20 nm (Figure 11c).

### 4.4. Oscillating Barrier Method and Compression Isotherms

The dynamic surface properties were measured by the oscillating barrier method. The oscillatory motion movement of two barriers along the brims of a Langmuir trough leads to periodical changes in the surface area. The induced surface tension oscillations of the liquid in the trough were measured by a Wilhelmy plate method. If the amplitude of the oscillations is sufficiently small, the dynamic surface elasticity *ε* can be determined by the ratio of the increments of the surface tension Δ*γ* and the relative changes in the surface area Δ*A/A*:

(1) $$\varepsilon =\frac{\Delta \gamma }{\Delta A/A}$$

In general, the dynamic surface elasticity is a complex quantity. In this work, the imaginary part was much smaller than the real one and almost coincided with the modulus of the dynamic surface elasticity. Therefore, only the modulus of dynamic surface elasticity is discussed below.

The measurements were performed by the ISR instrument (KSV NIMA, Helsinki, Finland) at a constant frequency of 30 mHz. Unless it is particularly specified, the relative surface area change was 3%. The Lissajous plots were obtained for equilibrium adsorption layers by applying different amplitudes of the surface area oscillations from 5 to 20%.

The compression isotherms were measured for equilibrium surface layers at a constant rate of the barrier motion of 10 mm/min.

### 4.5. Ellipsometry

The thickness of the surface layer and the surface coverage were calculated from the results of ellipsometric measurements based on recording the changes in the parameters of elliptically polarized light after reflection from the liquid surface [59,60]. The ratio of the amplitudes of the parallel and perpendicular components of the electric field of the light beam after reflection, normalized to their initial values, is described by the angle Ψ, and the phase shift between the incident and reflected waves is expressed by the angle Δ. The measurements were carried out using a Multiscop null ellipsometer (Optrel GBR, Berlin, Germany), where the light source was a helium-neon laser with a wavelength of 632.8 nm. The angle of incidence was 49°.

The obtained Ψ and Δ values are determined by the properties of the reflecting surface. In the model of a thin homogeneous layer between two bulk phases, these parameters can be related to the refractive index $n_{1}$ and thickness δ of the interfacial layer by the following equation [61]:

(2) $$tan\Psi e^{i\Delta }=tan\Psi_{0}e^{i\Delta_{0}}*\left[1+i\left(\frac{4\pi \delta cos\varphi_{0}{sin}^{2}\varphi_{0}n_{2}^{2}M}{\lambda \left(n_{2}^{2}-n_{0}^{2}\right)\left(n_{0}^{2}{sin}^{2}\varphi_{0}-n_{2}^{2}{cos}^{2}\varphi_{0}\right)}\right)\right]$$

where $\Psi_{0}$ and $\Delta_{0}$ are the ellipsometric angles for the bare interface, $n_{0}$ and $n_{2}$ are the refractive indices of bulk phases, $\varphi_{0}$ is the angle of incidence, λ is the light wavelength and $M=n_{0}^{2}+n_{2}^{2}-n_{1}^{2}-n_{0}^{2}n_{2}^{2}/n_{1}^{2}$.

The refractive index $n_{1}$ and layer thicknesses δ can be calculated on the basis of this equation using Ψ, $\Psi_{0}$, Δ, $\Delta_{0}$ by a numerical iteration method.

### 4.6. Atomic Force Microscopy

Near the steady state, the microscopic layer morphology was determined using atomic force microscopy (AFM) in the tapping mode of NTEGRA Spectra NT–MDT equipment (Zelenograd, Russia). The films were transferred from the aqueous surface to a freshly cleaved mica plate using the Langmuir-Schaeffer method. After that, the samples were dried in a desiccator for three days.

## 5. Conclusions

The surface properties of spread films of silk fibroin ALF and amyloid fibrils of globular proteins are similar. Both systems demonstrate an increase in the surface elasticity up to 140 mN/m, spread effectively on the surface of aqueous NaCl solutions, and depend strongly on the concentration of unreacted protein. At the same time, the dynamic surface elasticity of the layers of ALF is lower than that of the RSF layers. This is observed not only for spread ALF films but also for other systems containing fibroin ALF. It is shown that the main role in the reduction in the dynamic surface elasticity is played by ALF. Other factors, like the concentration of RSF, the concentration of UP, and high heterogeneity of the surface layer, have a smaller impact. If the layer contains ALF, the self-assembled structures, which are typical for RSF, are not formed, or are formed to a noticeably lesser extent. Unlike the RSF, which forms thick (up to 40 nm), rigid networks at the interface, the ALF forms thinner (7–15 nm) and more heterogeneous surface layers. Ellipsometry and the surface compression isotherms confirm that the fibril layers have a smaller surface concentration of amino acid residues, and these layers undergo a two-step reorganization under surface stress, unlike the RSF layers. Thus, the fibroin fibrils act as structural modifiers that suppress the interfacial self-organization of fibroin molecules, leading to thinner, less resilient but stable surface layers.

## Data Availability

The data presented in this study are openly available in the article.

## Supplementary Materials

The following supporting information can be downloaded at: https://www.mdpi.com/article/10.3390/ijms27083546/s1.
