# Supplementary material for: Silk Fibroin Aggregates at the Air–Water Interface: Amyloid-like Fibrils vs. Self-Assembled Networks
Source: Int J Mol Sci. 2026 Apr 16;27(8):3546. doi: 10.3390/ijms27083546 (PMC13115616; doi:10.3390/ijms27083546)
Supplement: Supplementary file 1 [file ijms-27-03546-s001.zip › ijms-4226074-supplementary.pdf]

Supplementary Materials

Silk Fibroin Aggregates at the Air–Water Interface: Amyloid-like Fibrils vs. Self-Assembled Networks

Olga Y. Milyaeva [1,*], Anastasiya R. Rafikova [1], Alina S. Koneva [1], Reinhard Miller [2], Giuseppe Loglio [3] and Boris A. Noskov [1]

[1] Department of Colloid Chemistry, St. Petersburg State University, Universitetsky pr. 26, 198504 St. Petersburg, Russia; nastya.rafikova.2000@mail.ru (A.R.R.); a.koneva@spbu.ru (A.S.K.); b.noskov@spbu.ru (B.A.N.)

[2] Institute of Condensed Matter Physics, Technische Universität Darmstadt, D-64289 Darmstadt, Germany; reinhard.miller@pkm.tu-darmstadt.de

[3] Institute of Condensed Matter Chemistry and Technologies for Energy, 16149 Genoa, Italy; giuseppe.loglio@ge.icmate.cnr.it

* Correspondence: o.milyaeva@spbu.ru; Tel.: +7-9062491297

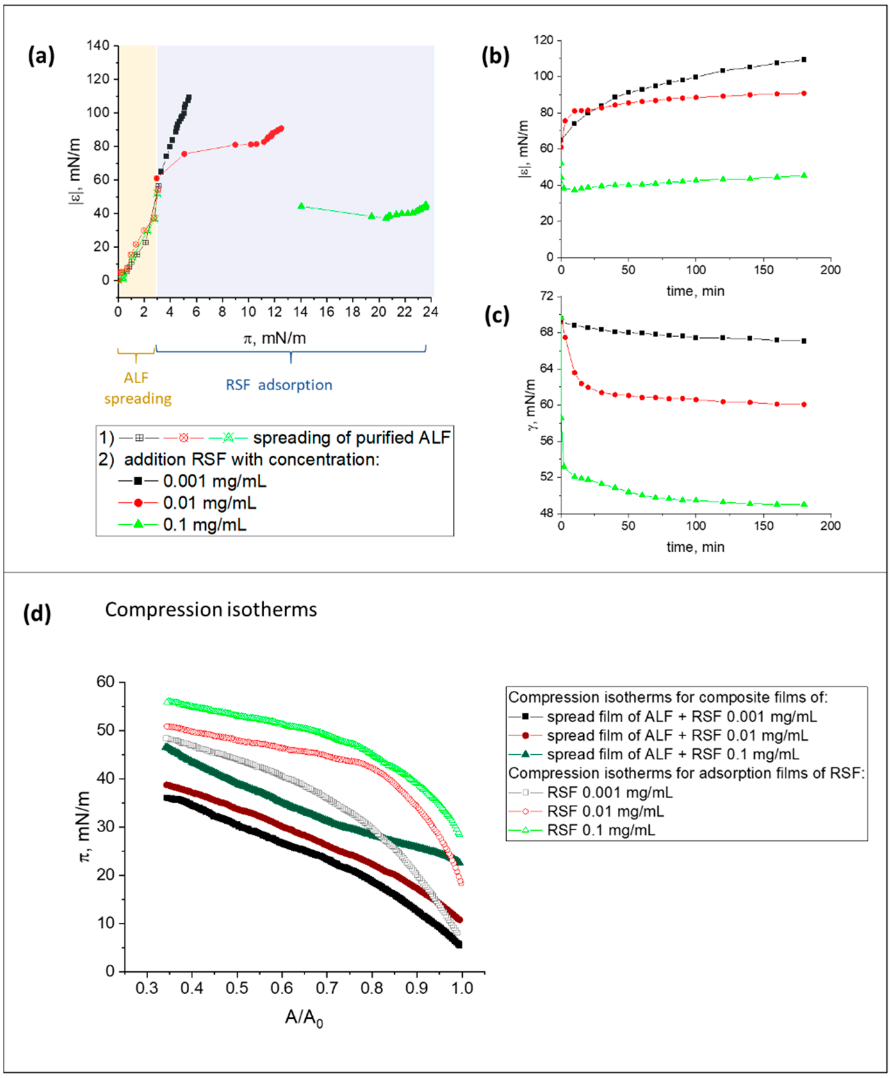

Figure S1. The dependencies of the dynamic elasticity modulus on surface pressure for composite films with different concentrations of the protein added to the bulk (**a**). The kinetic dependences of the dynamic surface elasticity (**b**) and dynamic surface tension (**c**) for composite films. The compression isotherms of the composite films and adsorption layers of native SF (**d**).

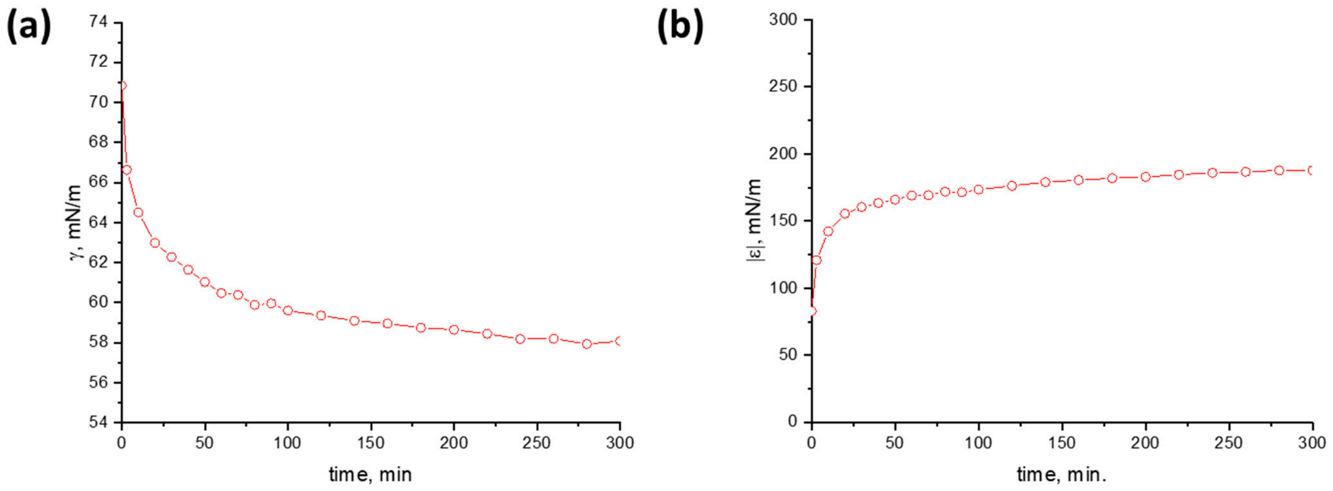

Figure S2. Kinetic dependences of (**a**) dynamic surface tension, (**b**) dynamic surface elasticity for isolated UP.

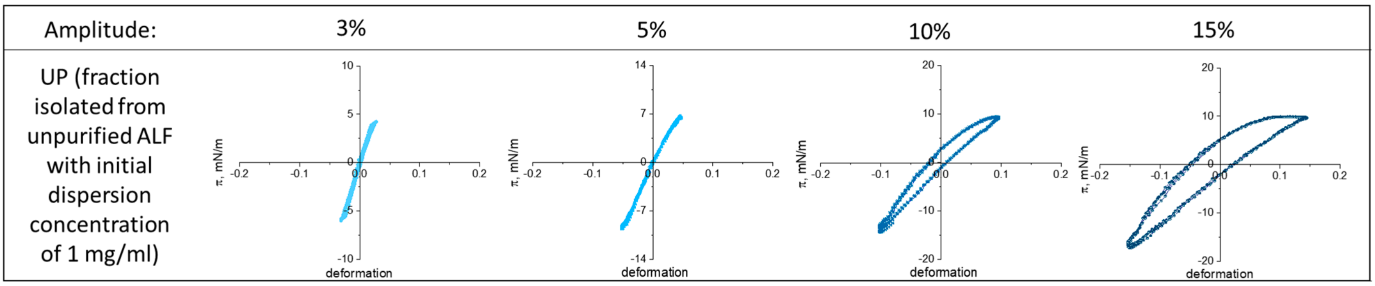

Figure S3. Lissajous plots of surface pressure versus deformation obtained during amplitude sweeps of adsorption layers of isolated UP.
